# HER3 functions as an effective therapeutic target in triple negative breast cancer to potentiate the antitumor activity of gefitinib and paclitaxel

**DOI:** 10.1186/s12935-023-03055-w

**Published:** 2023-09-16

**Authors:** Hui Lyu, Fei Shen, Sanbao Ruan, Congcong Tan, Jundong Zhou, Ann D. Thor, Bolin Liu

**Affiliations:** 1 Departments of Interdisciplinary Oncology and Genetics, Stanley S. Scott Cancer Center, School of Medicine, Louisiana State University (LSU) Health Sciences Center, 1700 Tulane Ave, New Orleans, LA 70112 USA; 2 Jiangsu Institute of Hematology, NHC Key Laboratory of Thrombosis and Hemostasis, National Clinical Research Center for Hematologic Diseases, The First Affiliated Hospital of Soochow University, Suzhou, Jiangsu China; 3 Suzhou Cancer Center Core Laboratory, Nanjing Medical University Affiliated Suzhou Hospital, Suzhou, Jiangsu China; 4 Department of Pathology, School of Medicine, University of Colorado Anschutz Medical Campus, Aurora, CO USA

**Keywords:** HER3, EGFR, Blocking antibody, Targeted therapy, Paclitaxel, TNBC

## Abstract

**Background:**

Triple negative breast cancer (TNBC) represents a significant clinical challenge. Chemotherapy remains the mainstay for a large part of TNBC patients, whereas drug resistance and tumor recurrence frequently occur. It is in urgent need to identify novel molecular targets for TNBC and develop effective therapy against the aggressive disease.

**Methods:**

Immunohistochemistry was performed to examine the expression of HER3 in TNBC samples. Western blots were used to assess protein expression and activation. Cell proliferation and viability were determined by cell growth (MTS) assays. TCGA databases were analyzed to correlate *HER3* mRNA expression with the clinical outcomes of TNBC patients. Specific shRNA was used to knockdown HER3 expression. IncuCyte system was utilized to monitor cell growth and migration. LIVE/DEAD Cell Imaging was to detect live and dead cells. HER3 recognition by our anti-HER3 monoclonal antibody (mAb) 4A7 was verified by ELISA, flow cytometry, and co-immunoprecipitation assays. Orthotopic tumor models were established in nude mice to determine the capability of TNBC cells forming tumors and to test if our mAb 4A7 could potentiate the antitumor activity of paclitaxel in vivo.

**Results:**

Elevated expression of HER3 was observed in approximately half of the TNBC specimens and cell lines tested. Analyses of TCGA databases found that the TNBC patients with high *HER3* mRNA expression in the tumors showed significantly worse overall survival (OS) and relapse-free survival (RFS) than those with low *HER3* expression. Specific knockdown of HER3 markedly inhibited TNBC cell proliferation and mammosphere formation in vitro and tumor growth in vivo. Our mAb 4A7 abrogated heregulin (a ligand for HER3), but not SDF-1 (a ligand for CXCR4)-induced enhancement of TNBC cell migration. Combinations of 4A7 and the EGFR-tyrosine kinase inhibitor (TKI) gefitinib dramatically decreased the levels of phosphorylated HER3, EGFR, Akt, and ERK1/2 in TNBC cells and potently induced growth inhibition and cell death. Moreover, 4A7 in combination with paclitaxel exerted significant antitumor activity against TNBC in vitro and in vivo.

**Conclusions:**

Our data demonstrate that increased HER3 is an effective therapeutic target for TNBC and our anti-HER3 mAb (4A7) may enhance the efficacy of gefitinib or paclitaxel in TNBC.

**Supplementary Information:**

The online version contains supplementary material available at 10.1186/s12935-023-03055-w.

## Background

Triple negative breast cancer (TNBC) represents a significant clinical challenge as the patients with TNBC have poor prognosis and account for a disproportionate number of breast cancer (BC) deaths [1–3]. It is defined as a BC subtype absence of estrogen receptor (ER) and progesterone receptor (PR) and lack of *HER2* amplification/overexpression [2, 4, 5], thus is unlikely driven by hormornal or HER2 signaling [6]. TNBC is more prevalent in African American women [7, 8], and associates with younger age, more advanced stage, higher rates of relapse and metastasis, and *BRCA* gene mutations [9, 10]. Although several targeted therapies, including PARP inhibitors [11–13] and Trop-2 antibody (Ab)-drug conjugate [14] as well as immunotherapy [15, 16] have been approved by FDA to treat advanced/metastatic TNBC, chemotherapy remains the standard of care for a large part of TNBC patients. However, drug resistance and tumor recurrence frequently occur, suggesting that TNBC is highly heterogeneous and it is in urgent need to identify novel targets and develop effective treatments for the aggressive disease [17, 18].

The human epidermal growth factor receptor (HER) family includes the epidermal growth factor receptor (EGFR, also known as HER1 or erbB1), HER2 (erbB2), HER3 (erbB3), and HER4 (erbB4). It is arguably the most characterized receptor tyrosine kinase (RTK) family contributing to both normal cell development and tumorigenesis [19, 20]. The HER receptors are commonly overexpressed in human cancers and play important roles in tumor initiation and progression [21, 22]. Among the family members, HER3 is unique, as it has no or deficient kinase activity [23–25]. HER3 must form heterodimers with another receptor to activate downstream signaling cascades promoting cell growth and survival. Studies on the underlying mechanisms demonstrate that activation of HER3-initiated signaling facilitates tumor progression mainly through enhanced metastatic potential of cancer cells and induced resistance to treatments [26–29]. HER3 is now recognized as an attractive target, and it is believed that effective inactivation of HER3 shall be able to overcome drug resistance and increase therapeutic efficacy and patient survival [26, 30–32]. Indeed, a number of HER3-targeted mAbs and Ab-drug conjugates (ADCs) have been reported to exert potent antitumor activity in preclinical studies of various cancer types and some of them show promising results in clinical evaluations [26, 30, 33, 34]. To date, however, there is no FDA-approved HER3-targeted therapy for human cancers. As HER3 expression is a major cause of cancer treatment failure [35, 36], majority of the current studies focus on defining the molecular basis of HER3 signaling-mediated therapeutic resistance in a wide variety of cancers, including HER2-positive breast cancer [28, 31], castration-resistant prostate cancer [37], platinum-resistant/refractory ovarian cancer [38, 39], and EGFR-TKI resistant NSCLC [40–43]. Nonetheless, our understanding of the unique biology of HER3 in TNBC is limited. It is not clear if HER3 can serve as a molecular target for TNBC. Interestingly, recent studies suggest that HER3 is a poor prognostic marker in hormone receptor-negative BC [44]. Examination of HER3 and EGFR protein levels shows that combined HER3-EGFR high score significantly associates with poor clinical outcomes in TNBC patients [45]. In the current studies, we have examined the expression of HER3 in TNBC specimens and cell lines, and performed bioinformatics analyses of TCGA databases to correlate *HER3* mRNA expression with the clinical outcomes of TNBC patients. We have also investigated the effects of specific knockdown of HER3 on TNBC cell proliferation in vitro and tumor growth in vivo, and explored whether our anti-HER3 mAb 4A7 would potentiate the antitumor activity of gefitinib and/or paclitaxel against TNBC.

## Methods

### Reagents and antibodies

Heregulin β1 (HRGβ1) and SDF-1 were purchased from R&D Systems, Inc. (Minneapolis, MN). Gefitinib and paclitaxel were from Selleck Chemicals, Inc. (Houston, TX). Antibodies used for western blot assays were from the following: HER3 (12,708), p-HER3 (Y1286), EGFR (4267), p-EGFR (Y1068), p-Akt (S473), Akt (9272), IGF-1R (3027), PARP (46D11), caspase-8 (1C12), caspase-3 (8G10) (Cell Signaling Technology, Inc., Danvers, MA); β-actin mouse mAb (clone AC-75) (Sigma-Aldrich, Inc., St. Louis, MO). Antibodies used for immunoprecipitation assays were from the following: HER3 (12,708), EGFR (4267), mouse IgG1 isotype control (5415), and rabbit IgG (2729) (Cell Signaling Technology, Inc.). Antibodies used for flow cytometry analysis were mouse anti-HER3 mAb (10,201-MM01) from Sino Biological, Inc. (Beijing, China). APC-labelled goat anti-mouse IgG Ab (405,308) from Biolegend Co. (San Diego, CA). All other reagents were from Sigma-Aldrich, Inc. unless otherwise specified.

### Cells and cell culture

Human breast cancer cell lines (SKBR3, MDA-MB-468, HCC70, HCC1806, MDA-MB-231, HCC1937, BT549, and Hs578T) were from the American Type Culture Collection (ATCC, Manassas, VA). The HER3-overexpressing subline SKBR3.B3.1, derived from SKBR3, was described previously [46, 47]. Cells were authenticated with DNA profiling by Short Tandem Repeat (STR) analysis in 2016–2018. The MycoAlert™ Mycoplasma Detection Kit (Lonza Group Ltd. Basel, Switzerland) was used to detect mycoplasma once every six months. All cell lines were free of mycoplasma contamination. Cells were maintained in DMEM/F-12 (1:1) medium (Sigma) supplemented with 10% fetal bovine serum (FBS) (Sigma) and cultured in a 37 °C humidified atmosphere containing 95% air and 5% CO2 and split twice a week.

### Cell growth/viability assay

Cell growth/viability was measured by MTS assays with CellTiter 96 Aqueous One Solution kit (Promega, Madison, WI) as described previously [47–49]. Cells were seeded in 96-well plates and treated with the relevant agents. Control cells were treated with vehicle. Absorbance was measured at 72 h after treatment.

### Examination of cell proliferation and migration

The IncuCyte™ system (Essen BioScience, Inc., Ann Arbor, MI) was used to kinetically monitor cell proliferation and migration. This automated imaging platform provides real-time images and quantitative data generated throughout the entire cell culture process. Proliferation was measured using a phase-only processing module as described previously [50]. Briefly, cells were plated onto 96-well plates with different treatments described in the figure legends. The plates were placed into the IncuCyte system at 37 °C for 2–3 days. During this time of period, each well was repeatedly scanned and imaged at fixed time intervals (every 4 h). The data were analyzed by the IncuCyte software. For migration assay, cells were seeded in IncuCyte® ImageLock plates. The following day, cells were treated with Mitomycin C (5 µg/ml) to inhibit cell proliferation for 2 h before using IncuCyte WoundMaker 96-pin tool to create cell-free zones with the touch of a button in cell monolayers. The cells were then cultured in medium containing different treatments described in the figure legends. Wound closure was visualized and analyzed in real-time inside the incubator with the IncuCyte® live cell analysis system and software. Data reflects the means of three independent experiments.

### Generation of anti-HER3 mAb 4A7

Our anti-HER3 mAb 4A7 was generated using hybridoma technology via immunization of BALB/C mice [51, 52]. In brief, 6–8 weeks old female BALB/C mice were purchased from SLAC Laboratory Animal Co., Ltd. (Shanghai, China) and maintained according to the protocol approved by the Institutional Animal Care and Use Committee (IACUC) at the First Affiliated Hospital of Soochow University. The mice were immunized via intraperitoneal injection of 1 × 10^7^ SKBR3.B3.1 cells four times (once every two weeks). The mice were then boosted via intravenous injection of 5 × 10^6^ SKBR3.B3.1 cells. Three days later, the mice were sacrificed, and their spleen cells were obtained for cell fusion with SP2/0 murine myeloma cells in a 5 to 1 ratio using polyethylene glycerol 1450. Antibody-secreting hybridomas were initially screened by cell based ELISA with SKBR3.B3.1 cells as positive selection and MDA-MB-231 cells as negative selection. Hybridoma 4A7 was chosen based upon the ELISA results and carried out limiting dilution assays to isolate a single clone, producing mAb named 4A7. Specificity of 4A7 was further determined by ELISA with human HER3 His Tag (ER3-H5223) protein (ACROBiosystems, Newark, DE).

### Production and purification of mAb 4A7

8–10 weeks old female BALB/c mice were pretreated with 0.5 mL of pristane. One week later, 5 × 10^6^ hybridoma 4A7 cells were intraperitoneal inoculated into the mice to generate ascites. The ascites fluid was collected once every 3 to 4 days for a total of 4 times per mouse. The fluid was centrifuged to remove cells and debris and then purified using protein A affinity chromatography. After elusion with sodium citrate buffer, the purified antibody was dialyzed extensively in PBS at 4 °C, sterile filtered with a 0.22-µm Millipore filter, and then stored at -80 C after freeze-dried.

### Flow cytometric analysis

Flow cytometric analyses were performed to detect Ab binding to membrane HER3. In brief, SKBR3.B3.1 cells grown in culture were harvested by trypsinization and washed with 0.1% bovine serum albumin in PBS. The cells resuspended in PBS (2 × 10^5^ cells/ml) were incubated with an Ab (4A7 or the anti-HER3 mAb (10,201-MM01)) on ice in the dark for 30 min, followed by another 30 min incubation with secondary APC-labeled goat anti-mouse IgG Ab (405,308). The samples were analyzed on a Beckman Coulter CytoFLEX flow cytometer and the data were processed using the Flowjo software (FLOWJO, Ashland, OR).

### Immunoprecipitation and western blot analysis

Immunoprecipitation and western blot assays were performed as described previously [53, 54]. In brief, equal amounts of total cell lysates were incubated with primary Ab for 2 h at 4^o^C, followed by incubation with protein A or G-agarose (Sigma-Aldrich) at 4^o^C overnight. The immunoprecipitates or equal amounts of cell lysates were boiled in SDS-sample buffer, resolved by SDS-PAGE, transferred to nitrocellulose membrane (Bio-Rad Laboratories, Hercules, CA), and probed with the primary Ab described in figure legends. After the blots were incubated with horseradish peroxidase-labeled secondary Ab (Jackson ImmunoResearch Laboratories, West Grove, PA), the signals were detected using the enhanced chemiluminescence reagents (GE Healthcare Bio-Sciences Corp., Piscataway, NJ).

### Mammosphere formation assay

Mammosphere formation assays were performed to detect self-renewal capacity of cancer stem cells. Briefly, 2000 TNBC cells were plated in ultralow attachment plates (Corning Costar Corp., Cambridge, MA). Cells were grown in serum-free MammoCult basal medium (StemCell Technologies Inc, Vancouver, BC), plused with MammoCult Proliferation supplement and 1 ng/ml Hydrocortisone (Invitrogen) in 6-well Ultra Low Attachment plates (Corning Inc., Corning, NY). The medium was replenished every 3 days. After 14 days of incubation, the mammospheres (equal or greater than 50 micrometer in diameter) were counted.

### Live/dead cell staining assay

Cells were seeded in a 24-well plate overnight. After treatment, cells were stained with the LIVE/DEAD Cell Imaging kit (488/570) (Thermo Fisher Scientific, Inc., Waltham, MA) as described previously [49]. After incubation at room temperature in the dark, the cells were observed under EVOS FLoid Cell Imaging System (Thermo Fisher Scientific) and measured for live cells (green) with Green-Light Channel and dead cells (red) with Red-Light Channel.

### Orthotopic tumor model

Athymic nu/nu female mice were purchased from Charles River Laboratories Inc. (Wilmington, MA) and maintained according to the procedures and guidelines approved by the Institutional Animal Care and Use Committee (IACUC) at School of Medicine, University of Colorado Anschutz Medical Campus. Luciferase-labelled HCC1806 cells (5 × 10^5^) were suspended in 100 µL of PBS, mixed (1:1) with Matrigel (BD Biosciences, Franklin Lakes, NJ), and orthotopically inoculated into the mammary fat pads of the mice to establish orthotopic tumors. Mice were imaged by a bioluminescence IVIS imaging system once a week to monitor tumor growth. Tumor formation was also measured with fine calipers twice a week. The tumor volume was calculated by the formula: Volume = (Length × Width^2^)/2, where length was the longest axis and width the measurement at a right angle to the length. When tumor volume reached ~ 80mm^3^, the tumor-bearing mice received intraperitoneal injections of PBS, 4A7 (20 mg/kg), paclitaxel (6 mg/kg), or both 4A7 and paclitaxel (n = 5). The tumor growth curves were plotted using average tumor volume and followed by statistical analysis as we described previously [47, 49, 55]. At the end of the experiments (after 6 treatments), all mice were sacrificed. The mammary tumors were dissected and imaged by a digital camera.

### Immunohistochemistry (IHC) analysis

IHC assays were performed as described previously [48, 49]. HER3 expression in clinical samples was examined in a tissue microarray (TMA) (BR1301, US Biomax, Inc., Rockville, MD) consisting of 125 cases of TNBC specimens, whose characteristics, including pathology grade, TNM, clinical stage, and IHC (ER, PR, HER2) results are available online (BR1301 Tissue Array and Tissue Microarray of premade types). The TMA slides were deparaffinized and heated at 95^o^C for 30-40 min in Borg Decloaker, RTU (Biocare Medical, Pacheco, CA) for antigen retrieval, then blocked with a blocking sniper (Biocare Medical, Pacheco, CA), and then incubated with the anti-HER3 rabbit mAb (Cell Signaling Technology, cat# 12,708, 1: 400 dilution) at room temperature for 1 h. After washing with Tris Buffer Saline (pH 8.0), the slides were incubated with MACH 1 HRP Polymer detection kit (Biocare Medical) according to the manufacture’s instruction. The staining colors were developed with a DAB Chromogen Kit (Biocare Medical). Finally, all sections were counterstained in Mayer’s hematoxylin, nuclei blued in 1% ammonium hydroxide (v/v), dehydrated, and then mounted with permanent aqueous mounting medium (Bio-Rad). Digital images were captured using a Nikon Microscope. IHC staining was scored as the following: Score 0: no staining. Score 1+: weak and incomplete membrane staining in less than 10% of the invasive tumor cells. Score 2+: weak and complete staining of the membrane in more than 10% of invasive cancer cells. Score 3+: strong and complete homogenous membrane staining in more than 30% of the invasive tumor cells. All IHC slides were reviewed by two individuals, including our pathologist, Dr. Ann Thor. Image J and Image J plugin IHC profiler were applied to quantify IHC analysis. The mean intensity of indicated protein was measured using Image J. Three fields of each group were assessed.

### Statistical analysis

Experimental data were statistically analyzed using two-sided Student’s t tests and two-way ANOVA tests. Significance was set at the P < 0.05. All values are reported at the mean +/- SD from at least three independent experiments.

## Results

### Elevated expression of HER3 is observed in TNBC samples and cell lines and significantly associates with poor prognosis in TNBC patients

**Fig. 1:**
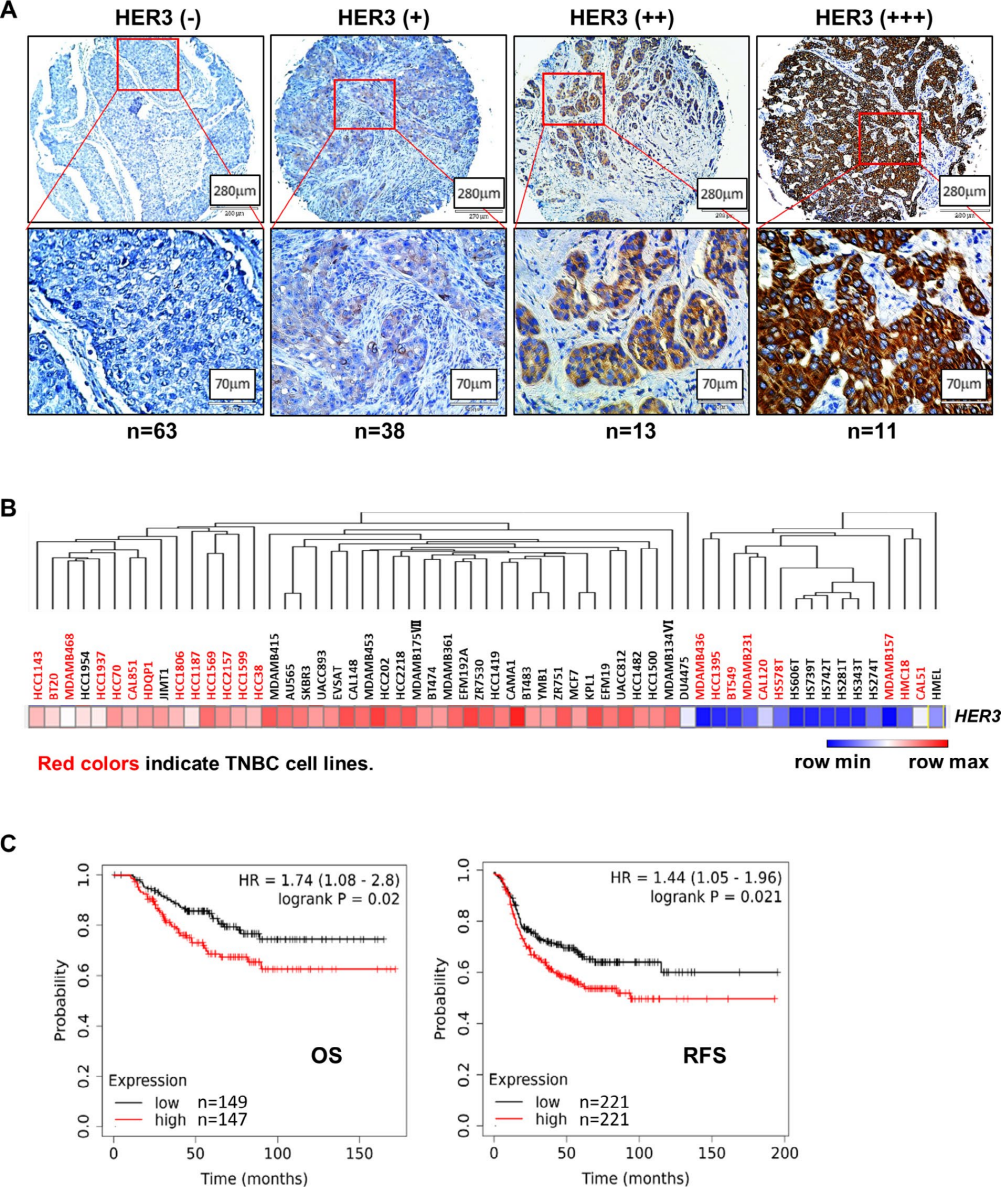
Elevated expression of HER3 is frequently observed in TNBC clinical samples and cell lines and associates with poor clinical outcomes in TNBC patients. **A**, IHC assays were performed to examine HER3 protein expression in a tissue microarray (TMA) of TNBC specimens (n = 125). Representative images of negative (-), low (+), medium (++), and high (+++) staining intensity of HER3 expression were shown. The case number in each group was shown underneath. **B**, The heatmap showed *HER3* mRNA expression in CCLE breast cancer cell lines (http://software.broadinstitute.org/morpheus). Red colors indicated TNBC cell lines. **C**, Analyses of overall survival (OS) or relapse-free survival (RFS) of basal-like breast cancer patients were performed using Kaplan-Meier Plotter (https://kmplot.com/analysis/). The expression levels of *HER3* mRNA were determined in TCGA-curated basal-like breast cancer datasets and used for log rank tests to compare the survival curves of those with high (red) or low (black) *HER3* expression

To determine whether HER3 may serve as a molecular target for TNBC treatment, we first examined the expression of HER3 in TNBC clinical samples. IHC analysis of a tissue microarray (TMA) consisting of 125 TNBC specimens showed that the expression of HER3 was observed in approximately half of the samples. In particular, HER3 was found to be highly expressed (HER3++/+++) in 24/125 (19.2%) TNBC cases (Fig. 1A). Next, we analyzed the Cancer Cell Line Encyclopedia (CCLE) databases to detect HER3 expression in human breast cancer cell lines. While 13 of the TNBC cell lines exhibited high *HER3* mRNA expression, the other 9 TNBC lines had little *HER3* mRNA expression (Fig. 1B). To assess the clinical significance of HER3 expression in TNBC, we then performed bioinformatics analyses of The Cancer Genome Atlas (TCGA) datasets to evaluate if *HER3* mRNA expression would correlate with the clinical outcomes of breast cancer patients. We found that the patients with basal-like tumors with high *HER3* mRNA expression significantly correlated with worse overall survival (OS) and relapse-free survival (RFS) as compared to that with low *HER3* expression (Fig. 1C). Thus, our data reveal that elevated expression of HER3 is frequently observed in TNBC clinical samples and cell lines, and increased HER3 significantly correlates with poor prognosis in TNBC patients.

### Specific knockdown of HER3 expression inhibits TNBC cell proliferation in vitro and tumor growth in vivo

**Fig. 2:**
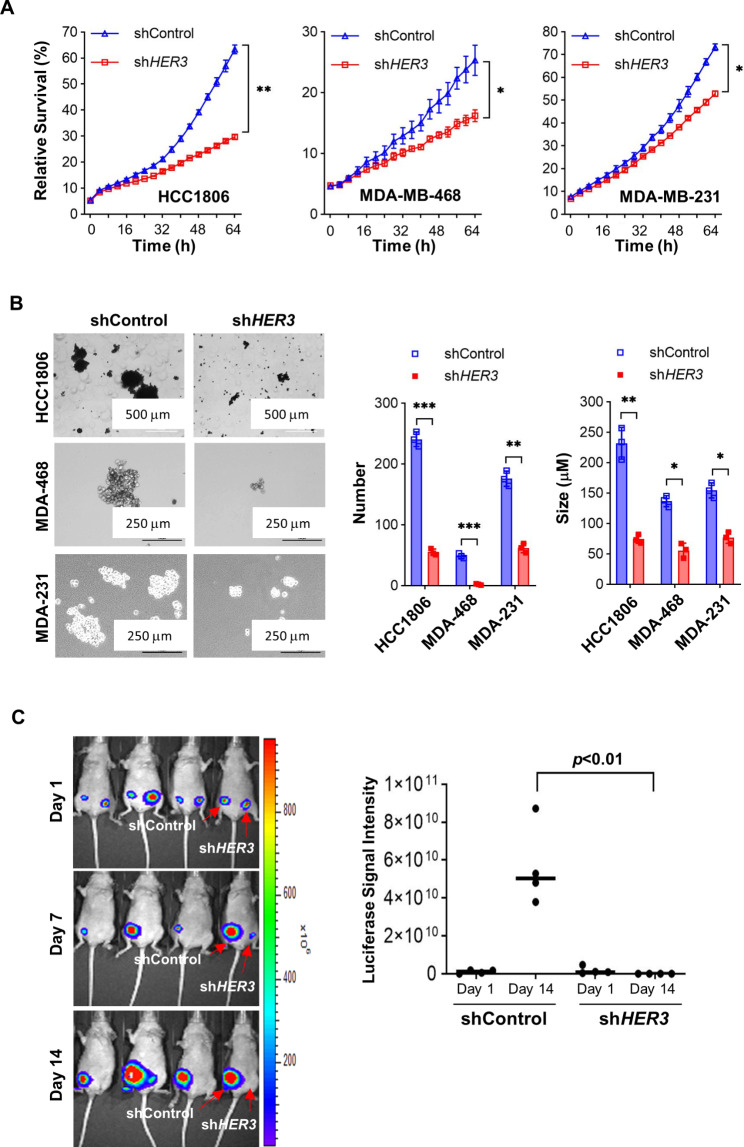
Specific knockdown of HER3 expression markedly inhibits TNBC cell proliferation and mammosphere formation in vitro and profoundly suppresses TNBC tumor growth in vivo. **A** and **B**, HCC1806, MDA-MB-468 (MDA-468), and MDA-MB-231 (MDA-231) cells were infected with lentivirus containing either control shRNA (shControl) or specific shRNA against HER3 (sh*HER3*) for 24 h. The cells were then seeded in 96-well plates, incubated at a regular cell culture incubator, and monitored for growth via an IncuCyte system for 64 h. The cell growth curves were generated by GraphPad Prism9 software with the data obtained through IncuCyte scanning every 4 h (**A**). The cells were then subjected to mammosphere formation assays. After 14 days, representative images of the mammospheres were shown and the number and size of those mammospheres were examined, quantified, and presented in the bar graphs (**B**). Data shows a representative of three independent experiments. Bars, SD. *, p < 0.05, **, p < 0.01, ***, p < 0.005. **C**, Luciferase-labelled HCC1806 cells were infected with lentivirus containing either control shRNA (shControl) or *HER3* shRNA (sh*HER3*) for 24 h. The HCC1806-shControl or HCC1806-sh*HER3* cells (5 × 10^5^) were orthotopically inoculated into the left or right mammary fat pads of female nude mice, respectively (n = 4). In vivo bioluminescent imaging of the mammary tumors was obtained with the IVIS spectrum at day 1, 7, or 14 after cell inoculation. The graph showed the luciferase signal intensity of the mammary tumors obtained from the mice injected with HCC1806-shControl or HCC1806-sh*HER3* cells at day 1 and 14. A significant difference was observed using a two-sided Student’s t-test

To provide direct evidence supporting the importance of HER3 in TNBC cell growth and survival, we took advantage of an *HER3*-targeting shRNA, which was proven to specifically downregulate HER3 in our previous reports [46, 48, 54, 56]. TNBC cells (HCC1806, MDA-MB-468, MDA-MB-231) infected with lentivirus containing either a control shRNA (shControl) or the *HER3*-targeting shRNA (sh*HER3*) were first anlyzed by western blot to verify knockdown of HER3 by the sh*HER3* we used (Supplementary Fig. 1). The cells were then examined by an IncuCyte System to generate real-time and quantitative data via kinetically monitoring cell growth. We discovered that specific knockdown of HER3 markedly inhibited proliferation in all three TNBC cell lines (Fig. 2A). Mammosphere formation assays showed that silencing of *HER3* significantly decreased the mammosphere number and size in HCC1806, MDA-MB-468, and MDA-MB-231 cells (Fig. 2B), suggesting that the expression of HER3 was critical to maintain the stem-like properties of TNBC cells. It seemed that specific knockdown of HER3 in TNBC (HCC1806 and MDA-468) cells with relatively high HER3 exprssion exhibited much more potent suppression on cell proliferation and mammosphere formation than that in TNBC (MDA-231) cells with low HER3 exprssion (Supplementary Fig. 1 to Fig. 2A & B). Moreover, we utilized mouse models to test the effects of HER3 manipulation on in vivo tumor growth. Luciferase-labelled HCC1806 (HCC1806-Luc) cells were infected with lentivirus containing either shControl or sh*HER3*. The resulting cells were orthotopically injected into the left or right mammary fat pads of female nude mice (n = 4), respectively. Bioluminescent imagining analyses of the tumors were obtained using IVIS Spectrum at day 1, 7, or 14 post-injection of the cells. We found that HCC1806-Luc cells transfected with sh*HER3*, as compared to those transfected with shControl, completely lost their capability to form tumors in nude mice (Fig. 2C), indicating that downregulation of HER3 substantially repressed TNBC tumor growth in vivo. Collectively, our studies demonstrate that inhibition of HER3 exerts significant antitumor activity against TNBC both in vitro and in vivo.

### Our newly developed anti-HER3 mAb 4A7 specifically abrogates HRGβ1-, but not SDF-1-mediated TNBC cell migration

**Fig. 3:**
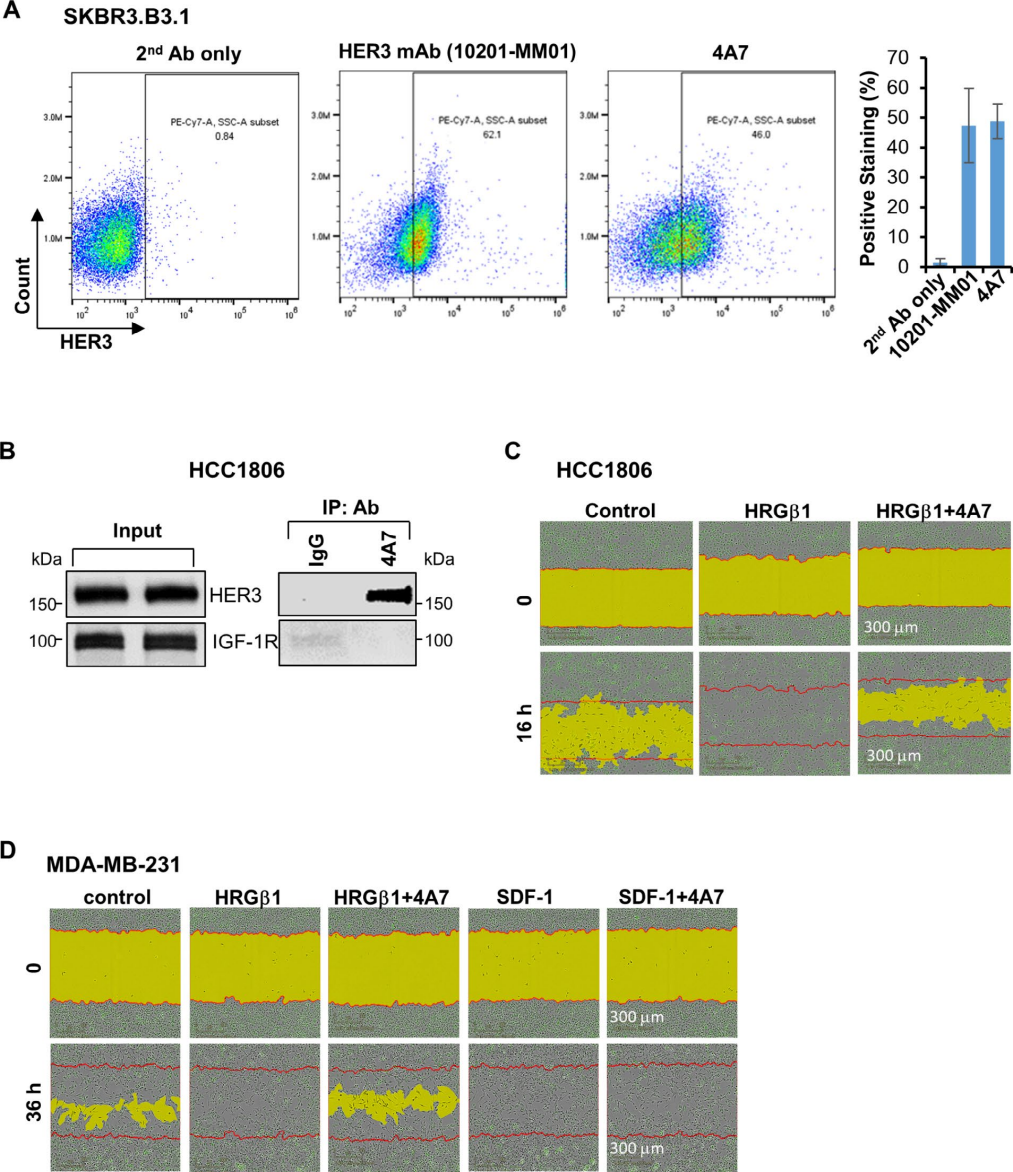
Our newly developed anti-HER3 mAb 4A7 recognizes cell membrane HER3, and exhibits profound activity to block HRGβ1-, but not SDF-1-induced TNBC cell migration. **A**, Our anti-HER3 mAb 4A7 was produced and purified as described in the materials and methods. 4A7 binding to membrane HER3 on SKBR3.B3.1 cells was examined by flow cytometry analysis. Staining with 2nd Ab only was used as a negative control. 10,201-MM01, a commercially available anti-HER3 mAb was used a positive control. Quantification of the positive staining with either 10,201-MM01 or 4A7 from three independent experiments was shown in the bar graphs. **B**, Equal amount of total protein extracts of HCC1806 cells were either examined by western blot analyses of HER3 and IGF-1R (Input) or subjected to IP (IP: Ab) using a control IgG or 4A7 and followed by western blot analyses of HER3 and IGF-1R. **C-D**, The specificity of 4A7 against HER3 was confirmed by its capability to block HRGβ1-induced promotion of TNBC cell migration. HCC1806 cells seeded in 96-well plates were pre-incubated with mitomycin C (2 µg/ml) prior to the scratch assays. The cells were then untreated or treated with HRGβ1 (50ng/ml) or HRGβ1 plus 4A7 (20ug/ml) and monitored by an IncuCyte system for cell migration (**C**). The same migration assays were performed with MDA-MB-231 cells in the presence of HRGβ1 (50ng/ml) or SDF-1 (200ng/ml) or their combinations with 4A7 (**D**)

The data described above suggest that HER3 has potential to be developed as a therapeutic target for TNBC. To date, no HER3-targeted therapy has been approved for cancer treatment. Due to its lack of or impaired kinase activity [23–25], targeting HER3 with a blocking Ab is the only approach currently being examined in pre-clinical and clinical evaluations [26, 30]. A number of HER3-targeted Abs, including mAb and Ab-drug conjugates (ADCs) have been shown to exhibit antitumor activity against a wide variety of cancer types [26, 30, 33, 34]. However, there is limited study testing the therapeutic potential of an anti-HER3 Ab in TNBC. We recently developed a mouse anti-human HER3 mAb named 4A7, which was purified from mouse ascites fluid. Flow cytometry analysis showed that 4A7 exhibited a similar efficiency as the commercial anti-HER3 mAb (10,201-MM01) to recognize membrane HER3 on SKBR3.B3.1 cells (Fig. 3A). Further studies with co-immunoprecipitation assays revealed that 4A7 specifically pulled down HER3, but not the insulin-like growth factor-1 receptor (IGF-1R) in HCC1806 cells (Fig. 3B). To verify 4A7’s specificity towards HER3 and examine if 4A7 would inhibit HER3 signaling, we performed wound-healing assays by taking advantage of the IncuCyte system kinetically monitoring cell migration. As compared to control, the HER3 ligand heregulin β1 (HRGβ1) clearly promoted migration in HCC1806 cells. In contrast, 4A7 abrogated HRGβ1-induced HCC1806 cell migration (Fig. 3C), suggesting HER3 inactivation by 4A7. Moreover, this 4A7-mediated blockade was specific for HRGβ1-, but not SDF-1 (stromal derived factor-1)-induced promotion of cell migration in MDA-MB-231 cells (Fig. 3D). Taken together, our anti-HER3 mAb 4A7 specifically recognizes and inactivates HER3, and potently abrogates HRGβ1-mediated TNBC cell migration.

### 4A7 in combination with gefitinib dramatically inactivates both HER3 and EGFR and induces potent anti-proliferative/anti-survival effects on TNBC cells

**Fig. 4:**
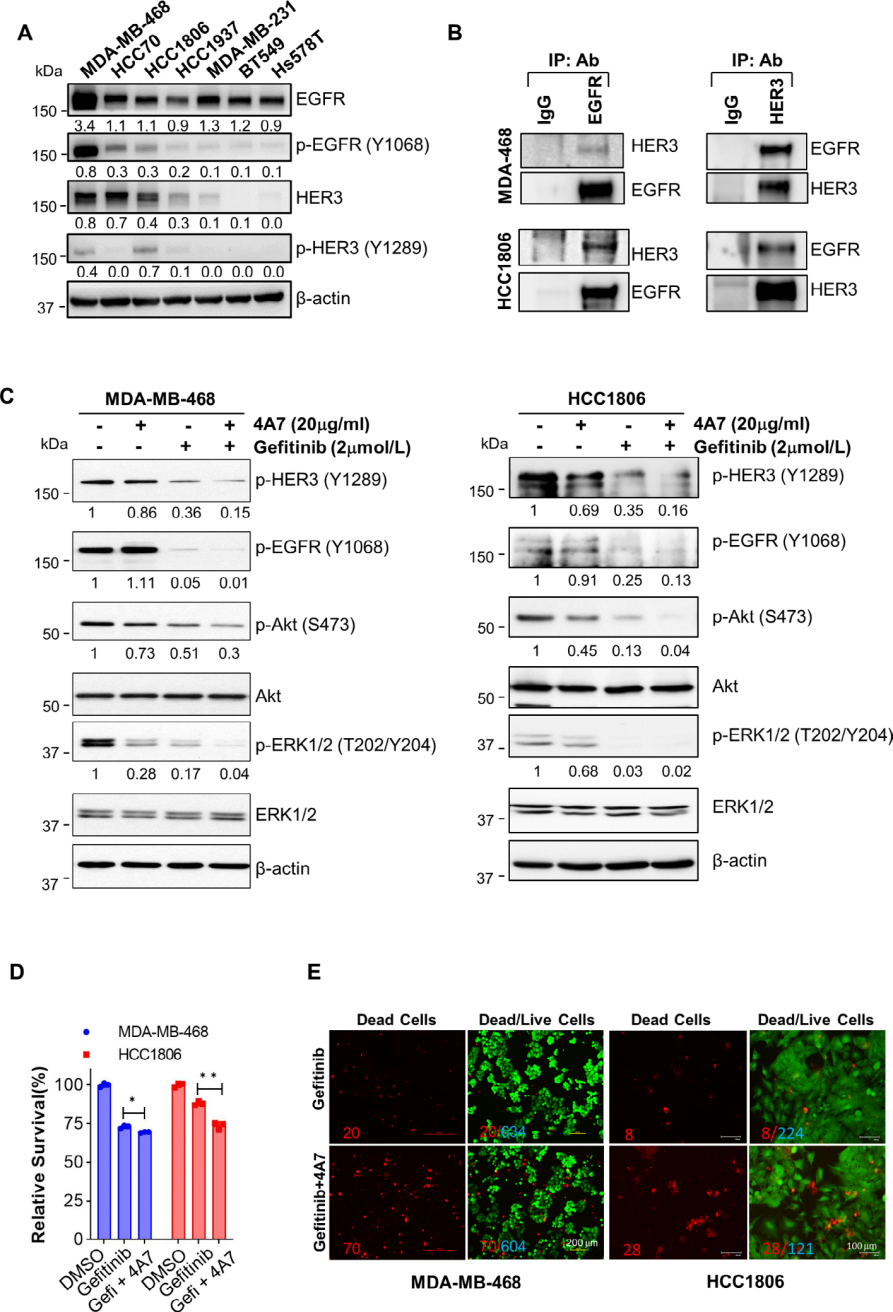
Combinations of 4A7 and gefitinib potently inactivate HER3, EGFR, and their downstream signaling, and markedly induce growth inhibition and cell death in TNBC cells. **A**, The indicated TNBC cell lines cultured at normal condition were collected and subjected to western blot analyses of EGFR, p-EGFR (Y1068), HER3, and p-HER3 (Y1289). β-actin was used as a loading control. The relative signal intensity obtained via densitometry analyses was shown underneath of each image. **B**, Equal amount of total protein extracts of MDA-MB-468 (MDA-468) or HCC1806 cells were subjected to IP using a control IgG, anti-EGFR Ab (left), or anti-HER3 Ab (right), and followed by western blot analyses of HER3 and EGFR. **C**, MDA-MB-468 or HCC1806 cells at normal culture condition were treated with vehicle, 4A7, gefitinib, or the combinations of 4A7 and gefitinib for 24 h. The cells were collected and subjected to western blot assays of the indicated markers. Densitometry analyses of the signals were shown underneath, and the arbitrary numbers indicated the intensities of each image relative to the untreated controls, defined as 1.0. **D**, MDA-MB-468 or HCC1806 cells were plated in 96-well plates. After 24 h, the culture medium was replaced with fresh medium containing vehicle control (DMSO), gefitinib (4µmol/L) alone, or gefitinib (4µmol/L) plus 4A7 (20 µg/ml) (Gefi + 4A7), and incubated for additional 72 h. The percentages of surviving cells relative to controls, defined as 100% survival, were determined by cell growth/viability (MTS) assays. Data shows a representative of three independent experiments. Bars, SD. *, p < 0.05, **, p < 0.01. **E**, MDA-MB-468 or HCC1806 cells seeded in 6-well plates were treated with gefitinib (8µmol/L) or its combinations with 4A7 (20 µg/ml) for 48 h. The cells were subjected to the LIVE/DEAD Cell Imaging assays. Green, live cells; red, dead cells. The ratio of dead/live cells was shown by in each sample. Data shows a representative of three independent experiments

In order to activate signaling pathways promoting cell proliferation and survival, HER3 has to form heterodimers with another receptor. As EGFR is one of the most preferred dimerization partners for HER3 [26] and commonly overexpressed in TNBC [57], we wondered if HER3 might interact with EGFR to form dimerization in TNBC cells with co-expression of EGFR and HER3. To this end, our western blot assays first confirmed EGFR overexpression in almost all TNBC cell lines tested (Fig. 4A). We also observed high expression of HER3 in about half of those TNBC cell lines, consistent with our findings in the examination of TNBC specimens and *HER3* mRNA expression (Fig. 1A & B). Next, we performed immunoprecipitation (IP)-western blot assays and detected strong interactions between HER3 and EGFR in MDA-MB-468 (MDA-468) and HCC1806 cells (Fig. 4B). It seemed that the HER3/EGFR interactions activated both receptors, evidenced by the high levels of phosphorylated EGFR (p-EGFR) and HER3 (p-HER3) (Fig. 4A). Thus, we hypothesized that TNBC cells with co-expression of EGFR and HER3 might be “addictive” to HER3/EGFR dimerization and activation for proliferation and survival, thereby being susceptible to the combinatorial treatment of an HER3-targeted therapy and an EGFR-targeted therapy. Indeed, our anti-HER3 mAb 4A7 in combination with the EGFR-TKI, gefitinib exhibited more dramatic effects than either 4A7 or gefitinib alone to reduce the levels of p-HER3, p-EGFR, p-Akt, and p-ERK1/2 in both MDA-MB-468 and HCC1806 cells (Fig. 4C). Moreover, combinations of 4A7 and gefitinib, as compared to gefitinib alone, significantly suppressed proliferation of MDA-MB-468 and HCC1806 cells (Fig. 4D). DEAD/LIVE Cell Imaging revealed that the combinations showed more potent activity than gefitinib to induce cell death in both MDA-MB-468 and HCC1806 cells (Fig. 4E). Collectively, our data indicate that 4A7 combined with gefitinib effectively inactivates HER3 and EGFR signaling and induces significant anti-proliferative/anti-survival effects on TNBC cells with expression of both EGFR and HER3.

### 4A7 significantly enhances paclitaxel-mediated antitumor activity against TNBC in vitro and in vivo

**Fig. 5:**
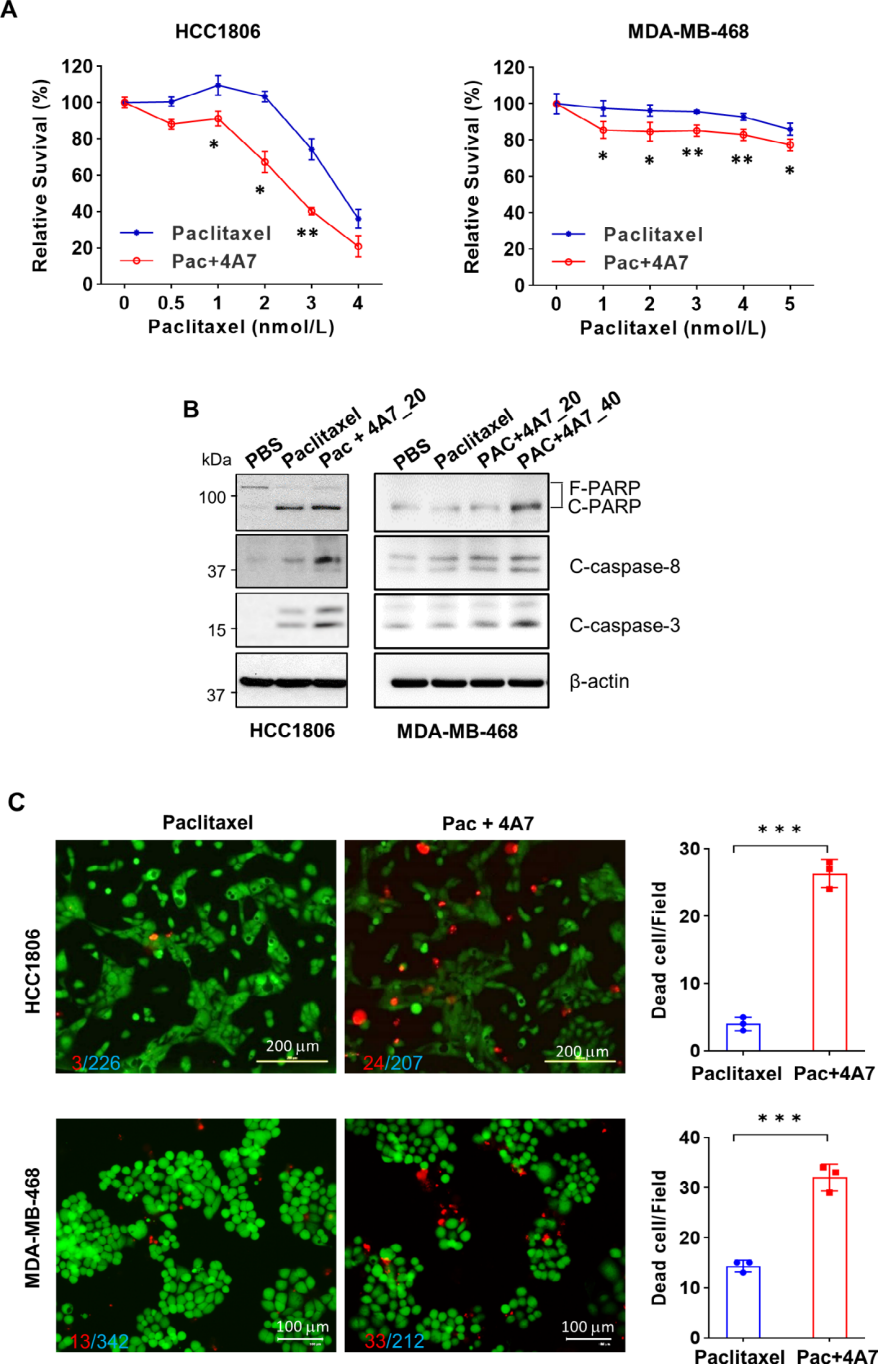
4A7 in combination with paclitaxel induces significant anti-proliferative and anti-survival effects on TNBC cells. **A**, MDA-MB-468 or HCC1806 cells were plated in 96-well plates. After 24 h, the culture medium was replaced with fresh medium containing indicated concentrations of paclitaxel with (Pac + 4A7) or without 4A7 (20 µg/ml), and incubated for additional 72 h. The percentages of surviving cells relative to controls, defined as 100% survival, were determined by MTS assays. Data shows a representative of three independent experiments. Bars, SD. *, p < 0.05, **, p < 0.01. **B**, MDA-MB-468 or HCC1806 cells were treated with vehicle (PBS), paclitaxel (2nmol/L), or combinations of 4A7 (20 or 40 µg/ml) and paclitaxel (2nmol/L) (Pac + 4A7) for 48 h. The cells were examined by western blot analyses of PARP (F-PARP, full length PARP; C-PARP, cleaved PARP), cleaved caspase-8 (C-caspase-8), cleaved caspase-3 (C-caspase-3), or β-actin. **C**, MDA-MB-468 or HCC1806 cells seeded in 6-well plates were treated with paclitaxel (4nmol/L) or combinations of 4A7 (20 µg/ml) and paclitaxel (4nmol/L) (Pac + 4A7) for 48 h. The cells were subjected to the LIVE/DEAD Cell Imaging assays. Dead cells were counted at three random fields. Bars, SD. ***, p < 0.0005

**Fig. 6:**
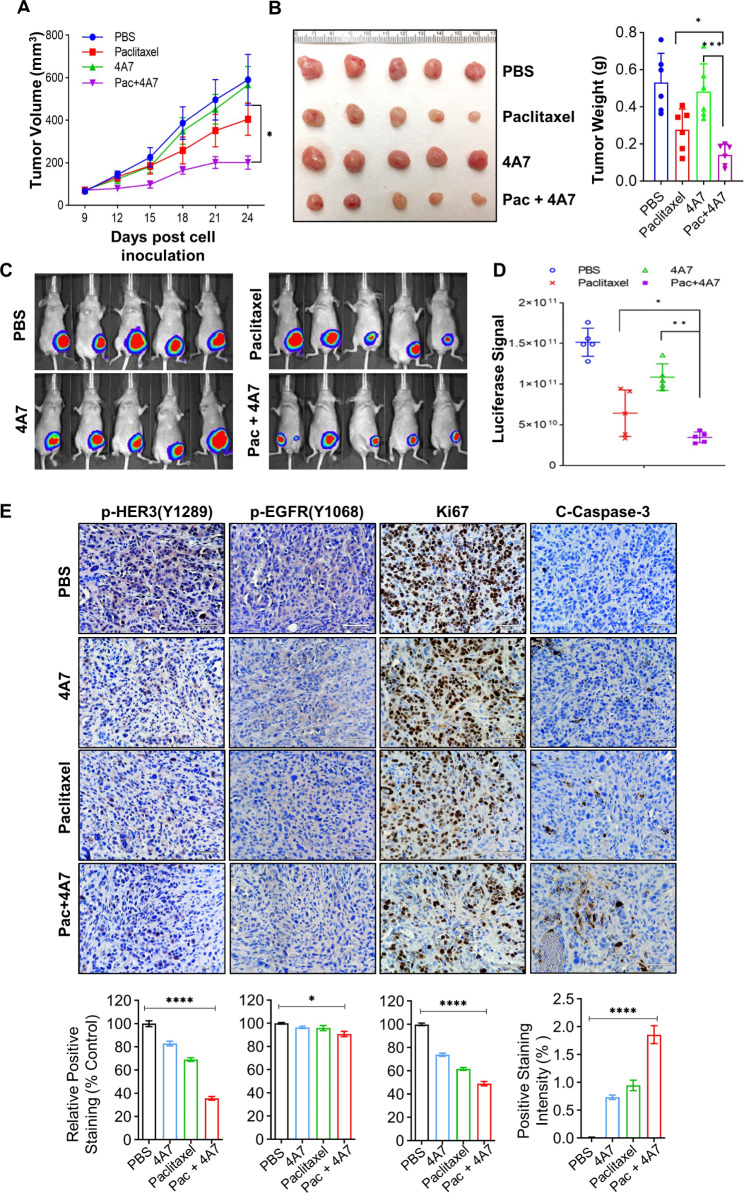
4A7 significantly enhances paclitaxel-mediated antitumor activity in TNBC tumor xenograft models. HCC1806-luc cells (5 × 10^5^) were orthotopically inoculated into the mammary fat pads of nude mice to establish tumor xenografts. When tumor volume reached ~ 80mm^3^, the tumor-bearing mice received intraperitoneal injections of PBS, 4A7 (20 mg/kg), paclitaxel (6 mg/kg), or both 4A7 and paclitaxel (Pac + 4A7) (n = 5). After 6 treatments, mice were sacrificed. **A**, Tumor growth curves were plotted using average tumor volumes within each group at the indicated time points. A two-tailed student’s t-test was used for statistical analysis. Bars, SEM. *, P < 0.01. **B**, The mammary tumors were dissected and imaged as indicated (Left). The tumors were also measured for weight (Right). Bars, SD. *, p < 0.05, ***, p < 0.005. **C** and **D**, At end of the experiments, before mice were sacrificed, bioluminescent imaging of the mammary tumors was obtained with the IVIS spectrum (**C**). The luciferase signal intensity of the mammary tumors from the mice of each treatment group was plotted. Bars, SD. *, p < 0.05, **, p < 0.01. **E**, Formalin-fixed paraffin-embedded sections of tumors were analyzed with IHC staining for p-HER3 (Y1289), p-EGFR (Y1068), Ki67, or cleaved Caspase-3 (C-caspase-3). Scale bar, 70 μm. Quantification of IHC staining with ImageJ and ImageJ plugin IHC profiler was shown underneath. A two-way ANOVA tests were used for statistical analysis. Bars, SD. *, p < 0.05, ****, p < 0.001

As HER3 has been implicated as a major cause of treatment failure [35, 36], effective inhibition of HER3 is believed to be required to overcome drug resistance and increase therapeutic efficacy and patient survival [26, 30–32]. It is conceivable to hypothesize that our anti-HER3 mAb 4A7 may potentiate the antitumor activity of a chemotherapeutic agent (paclitaxel) against TNBC. To test this hypothesis, we utilized both MDA-MB-468 and HCC1806 cells as our experimental models. In comparison with paclitaxel alone, combinations of 4A7 and paclitaxel significantly inhibited cell proliferation (Fig. 5A). The combinations were more effective than paclitaxel alone to induce PARP cleavage, which is the hallmark of apoptosis, and to increase cleaved caspase-8 and caspase-3 (Fig. 5B). DEAD/LIVE Cell Imaging confirmed that 4A7 in combination with paclitaxel, as compared with paclitaxel alone, significantly induced cell death in both HCC1806 and MDA-MB-468 cells (Fig. 5C). Furthermore, we examined if 4A7 could enhance the antitumor activity of paclitaxel against TNBC in vivo. HCC1806-Luc cells were orthotopically inoculated into the mammary fat pads of female nude mice to establish tumor xenografts. Tumor formation was assessed by palpation and measured with fine calipers twice a week. Tumor growth was also monitored by bioluminescent imaging once a week. When tumors reached ~ 80 mm^3^, the tumor-bearing mice were randomly assigned into four treatment groups (n = 5): PBS (control), 4A7, paclitaxel, and combinations of 4A7 and paclitaxel. All treatments were carried out via intraperitoneal injection twice a week for three weeks. We found that tumor growth curve in combination-treated mice was significantly slower than that in control or single agent-treated mice (Fig. 6A). While paclitaxel at the dose (6 mg/kg) we used exhibited minor inhibitory effects on tumor growth, 4A7 alone had no effect on tumor growth. These data were consistent with our previous studies with paclitaxel and another anti-HER3 mAb in HER2-positive breast cancer models [47]. At the end of this experiment, all mice were euthanized to obtain tumors for imaging (Fig. 6B). A clear reduction of tumor size was seen in the combination treatment group, confirming tumor growth inhibition. Bioluminescent imaging showed significantly weaker luciferase signal intensity in the mice treated with the combinations (Fig. 6C & D). There was no difference of the mouse bodyweight among the treatment groups (data not shown).

We next performed immunohistochemistry (IHC) analyses of the tumors to examine the treatment effects on HER3 and EGFR expression or activation as well as cell proliferation and apoptosis in vivo. Treatment with 4A7 or paclitaxel alone slightly, but the combinations of 4A7 and paclitaxel markedly reduced the levels of p-HER3. Although the reduction of p-EGFR levels seemed significant upon the combinatorial treatments, but to a much less extent (Fig. 6E). The treatments had no significant effects on HER3 and EGFR protein levels (Supplementary Fig. 2). Moreover, IHC analyses confirmed that combinations of 4A7 and paclitaxel as compared to either agent alone dramatically decreased the expression of Ki67, a classic cell proliferation marker, and increased the number of tumor cells with positive staining for cleaved caspase-3 (Fig. 6E). Collectively, our data demonstrate that targeting HER3 with 4A7 significantly potentiates paclitaxel-mediated antitumor activity against TNBC likely via potent inhibition of cell proliferation and enhanced apoptosis in vitro and in vivo.

## Discussion

HER3 emerges to be a critical factor causing drug resistance in human cancers and has now been recognized as an excellent target for cancer treatment [26, 28]. However, the unique biology of HER3 in TNBC progression remains elusive and there is limited study testing the therapeutic potential of an HER3-targeted therapy in TNBC. Here, we discovered an elevated expression of HER3 in approximately half of the TNBC clinical samples and cell lines tested, and high *HER3* expression significantly associated with poor OS and RFS in breast cancer patients with basal-like tumors (Fig. 1). By utilizing a genetic approach, we showed that silencing of *HER3* led to a remarkable suppression of TNBC proliferation in vitro and tumor growth in vivo (Fig. 2). Targeting of HER3 with our anti-HER3 mAb 4A7 not only increased the potency of the EGFR-TKI, gefitinib to inactivate both HER3 and EGFR and their downstream signaling, but also markedly potentiated gefitinib-mediated anti-proliferative/anti-survival effects on TNBC cells (Fig. 4). Moreover, 4A7 significantly enhanced the antitumor activity of paclitaxel against TNBC both in vitro and in vivo (Figs. 5 and 6). Thus, these proof of concept studies establish HER3 as an effective therapeutic target for TNBC. Our data indicate that an HER3-targeted therapy, including 4A7 has potential to be developed as a novel agent to enhance the efficacy of an EGFR-TKI or chemotherapy for TNBC treatment.

TNBC lacks *HER2* amplification/overexpression, thus is unlikely driven by HER2 signaling. In contrast, EGFR is overexpressed in majority of TNBC and elevated expression of EGFR significantly correlates with poor prognosis in TNBC patients [58, 59]. EGFR has been considered as an attractive target for TNBC [60, 61]. Yet, EGFR-targeted therapies, including mAbs and TKIs, show limited efficacy for TNBC treatment in both preclinical and clinical settings [62–64]. The disappointing data suggest that EGFR expression per se without considering its activation may not predict which TNBC patients benefit from EGFR-target therapy. Our studies revealed a strong interaction between HER3 and EGFR, presumably leading to activation of both receptors in the TNBC cells with co-expression of HER3 and EGFR (Fig. 4A & B), and these TNBC cells were highly sensitive to combinatorial treatments of 4A7 and gefitinib (Fig. 4C-E). Thus, assessment of the expression and activation (phosphorylation) of both HER3 and EGFR shall be more accurate to predict the sensitivity of TNBC to the combinations of an HER3-targeted therapy and an EGFR-TKI. Our idea is supported by a recent report showing that combined HER3-EGFR high score performs better than considering the receptors individually to predict worse clinical outcomes in TNBC patients [45]. Further investigations are warranted to determine the therapeutic potential of an anti-HER3 mAb in combination with an EGFR-targeted therapy in TNBC. In addition to EGFR, other RTKs, including MET (Mesenchymal-epithelial transition factor, also known as hepatocyte growth factor receptor (HGFR)), PDGFR (platelet-derived growth factor receptor), VEGFR (vascular endothelial growth factor receptor), insulin-like growth factor-1 receptor (IGF-1R), and fibroblast growth factor receptor (FGFR) are also commonly expressed in TNBC and have been identified as potential therapeutic targets [65–67]. While a number of TKIs against the RTKs have been approved by FDA for cancer treatment [68], the RTK-TKIs are largely ineffective in clinical trials of TNBC patients [65, 69], suggesting that at least some clones in TNBC tumors do not display dependency on the RTKs. Interestingly, HER3 has been shown to be able to interact with MET, IGF-1R, or FGFR3 in various human cancers [54, 70, 71]. Thus, it is conceivable to hypothesize that in TNBC, elevated expression of HER3 may utilize its unique biology to form heterodimers with a RTK, thereby activating the receptors and enabling the TNBC tumors acquiring ‘oncogenic addiction’ to HER3-RTK dimerization/activation. We are currently testing this hypothesis with multiple TNBC models. Data generated will not only identify HER3 as a potential biomarker predictive for the efficacy of RTK-TKIs in TNBC, but also facilitate the development of effective treatments for TNBC via rational combinations of an anti-HER3 mAb with a specific TKI of the RTK that interacts with HER3.

Although chemotherapy remains the mainstay for majority of TNBC and initially effective, drug resistance and tumor recurrence frequently occur [17]. It is critical to develop novel strategy to enhance chemotherapeutic efficacy in TNBC. Data presented here suggest that targeting of HER3 with a blocking Ab, including 4A7 is an effective approach to potentiate chemotherapy for the treatment of TNBC. We noticed that 4A7 or paclitaxel at low dose (6 mg/kg) alone had little effect on TNBC tumor growth, whereas their combinations significantly repressed tumor growth in vivo (Fig. 6). These findings were consistent with our previous studies with another anti-HER3 mAb, MM-121/seribantumab, which exerted potent antitumor activity against HER2-positive breast cancer only when MM-121 was combined with paclitaxel [47]. Activation of HER3 and its downstream signaling, especially the PI-3 K/Akt pathway can cause multidrug resistance [72, 73]. It is currently unclear if our anti-HER3 mAb 4A7 may be able to enhance the efficacy of other commonly used chemotherapeutic agents in TNBC, including doxorubicin, cyclophosphamide, 5-Fu, etc. The development of HER3 Ab-drug conjugates (ADCs) provides a new avenue to identify more effective HER3-targeted therapy for human cancers [26, 30, 74]. Recent reports from a phase I clinical trial reveal that the HER3 ADC (patritumab deruxtecan, U3-1402, or HER3-DXd) shows a good safety profile and promising clinical benefit in EGFR-TKI resistant NSCLC patients [75]. It will be interesting to test the antitumor activity of HER3-DXd in TNBC models.

In summary, we discovered that elevated expression of HER3 significantly associated with poor clinical outcomes in breast cancer patients with basal-like tumors. Further studies established HER3 as an effective therapeutic target for TNBC. Moreover, targeting of HER3 with our newly developed anti-HER3 mAb 4A7 not only potentiated gefitinib-mediated anti-proliferative/anti-survival effects on TNBC cells, but also significantly enhanced the antitumor activity of paclitaxel against TNBC both in vitro and in vivo. Our data indicate that an HER3-targeted therapy, including 4A7 has potential to be developed as a novel agent to enhance the efficacy of an EGFR-TKI or chemotherapy for TNBC treatment.

### Electronic supplementary material

Below is the link to the electronic supplementary material.

Supplementary Material 1

Supplementary Material 2

Supplementary Material 3

## Data Availability

All data generated for this study are included within this article and in the supplementary information. The data used and analyzed in the current study are available from the corresponding author on reasonable request.

## Abbreviations

TNBC: Triple negative breast cancer
RTK: Receptor tyrosine kinase
HER: Human epidermal growth factor receptor
EGFR: Epidermal growth factor receptor
ER: Estrogen receptor
PR: Progestrone receptor
FDA: Food and drug administration
TKI: Tyrosine kinase inhibitor
TMA: Tissue microarray
shRNA: Short hairpin RNA
Ab: Antibody
mAb: Monoclonal Ab
ADC: Ab-drug conjugate
MAPK: Mitogen-activated protein kinase
PI-3 K: Phosphoinositide 3-kinase
HRG: Heregulin
SDF-1: Stromal-derived factor-1
PARP: Poly (ADP-ribose) polymerase
IP: Immunoprecipitation
IHC: Immunohistochemistry
ELISA: Enzyme-linked immunosorbent assay
MTS: 3-(4,5-dimethylthiazol-2-yl)-5-(3-carboxymethoxyphenyl)-2-(4-sulfophenyl)-2 H-tetrazolium,inner salt
