# Supplementary material for: HER3 functions as an effective therapeutic target in triple negative breast cancer to potentiate the antitumor activity of gefitinib and paclitaxel
Source: Cancer Cell Int. 2023 Sep 16;23:204. doi: 10.1186/s12935-023-03055-w (PMC10504712; doi:10.1186/s12935-023-03055-w)
Supplement: Supplementary file 1 — Supplementary Material 1 [file 12935_2023_3055_MOESM1_ESM.docx]

**Supplementary Figure 1**

**
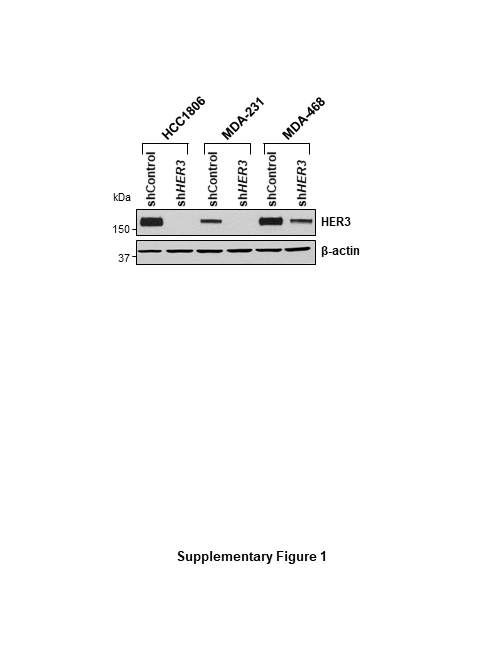
**

HCC1806, MDA-MB-231 (MDA-231), and MDA-MB-468 (MDA-468) cells were infected with lentivirus containing either control shRNA (shControl) or specific shRNA against HER3 (sh*HER3*) for 24 hours. The cells were collected and subjected to western blot analyses of HER3. β-actin was used as a loading control.

**Supplementary Figure 2**

**
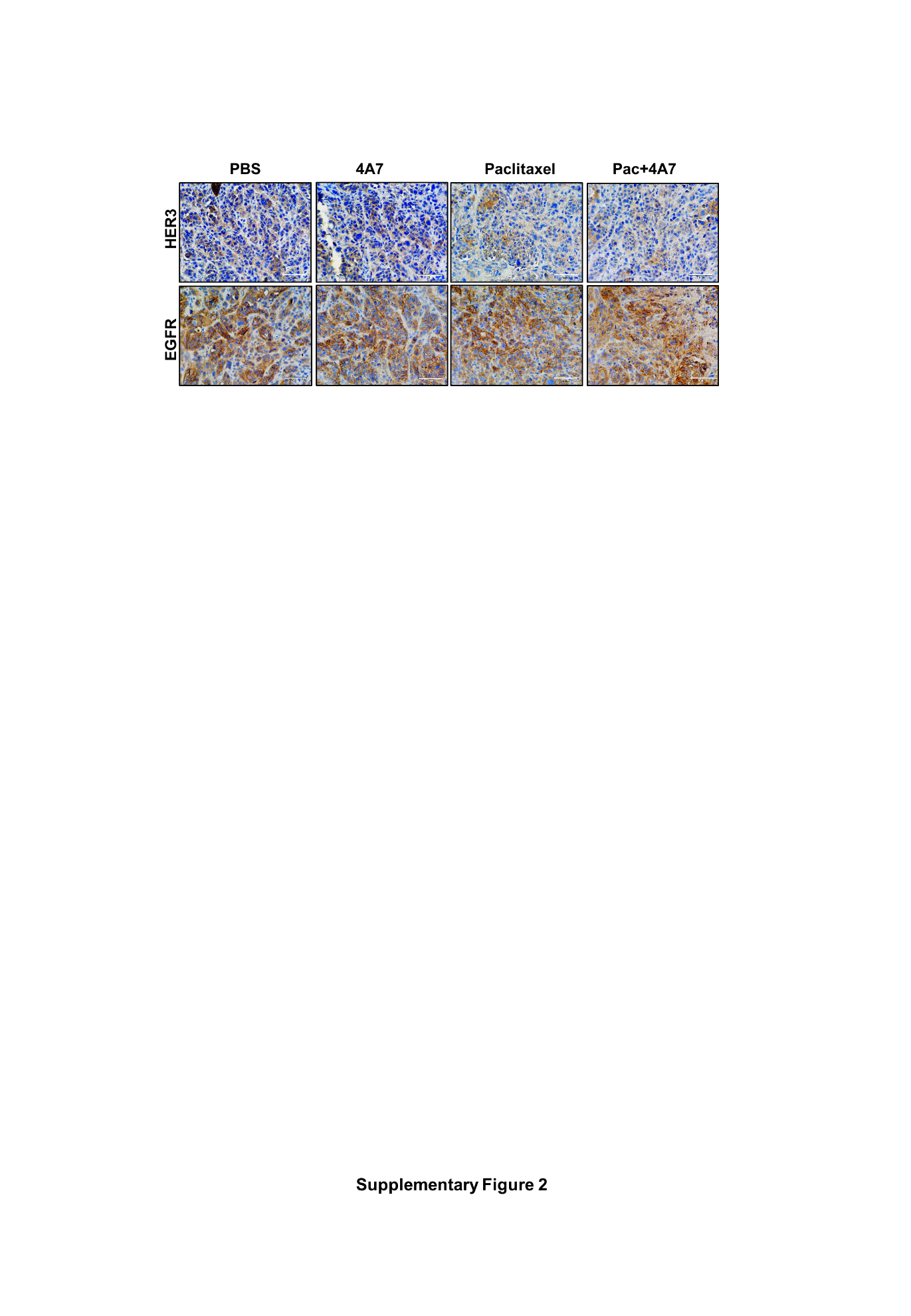
**

IHC analyses were performed to examine HER3 and EGFR expression in the tumors obtained from the animal experiments (related to Figure 6). No significant alterations were observed in the protein levels of both HER3 and EGFR with the treatment of either 4A7 or paclitaxel alone, or their combinations (Pac+4A7). Scale bar, 70µm.
