# Supplementary figures and images for: HER3 functions as an effective therapeutic target in triple negative breast cancer to potentiate the antitumor activity of gefitinib and paclitaxel
Source: Cancer Cell Int. 2023 Sep 16;23:204. doi: 10.1186/s12935-023-03055-w (PMC10504712; doi:10.1186/s12935-023-03055-w)

**Fig 3B**


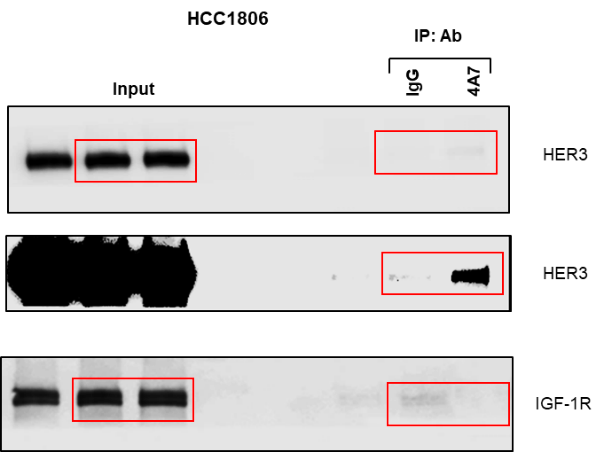


**Fig 4A**


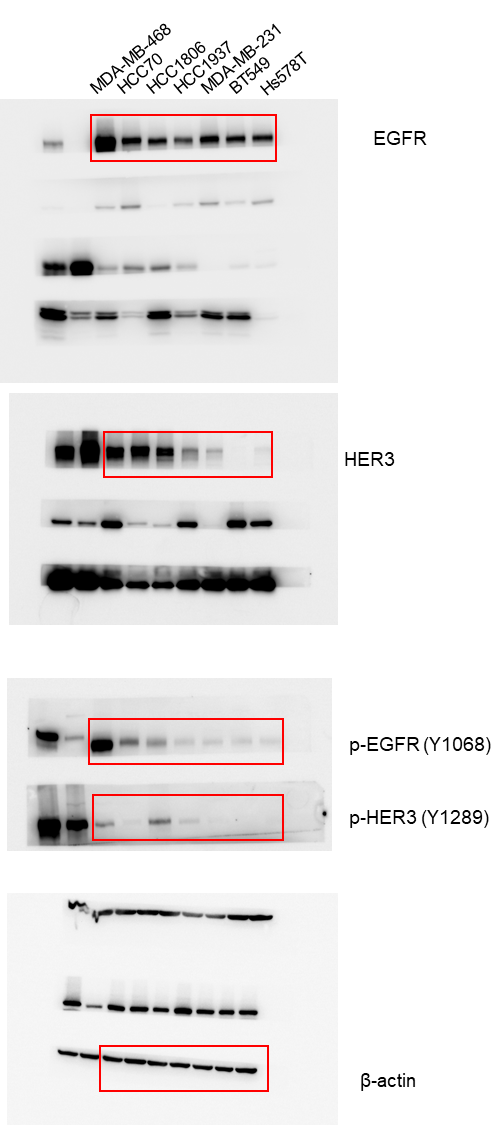


**Fig 4B**


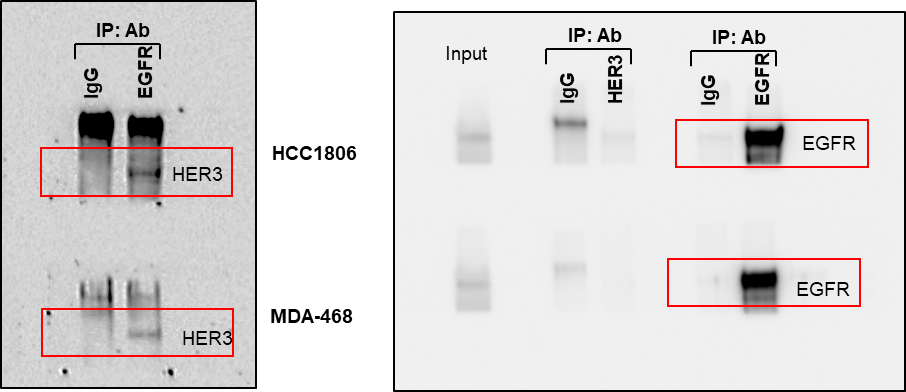


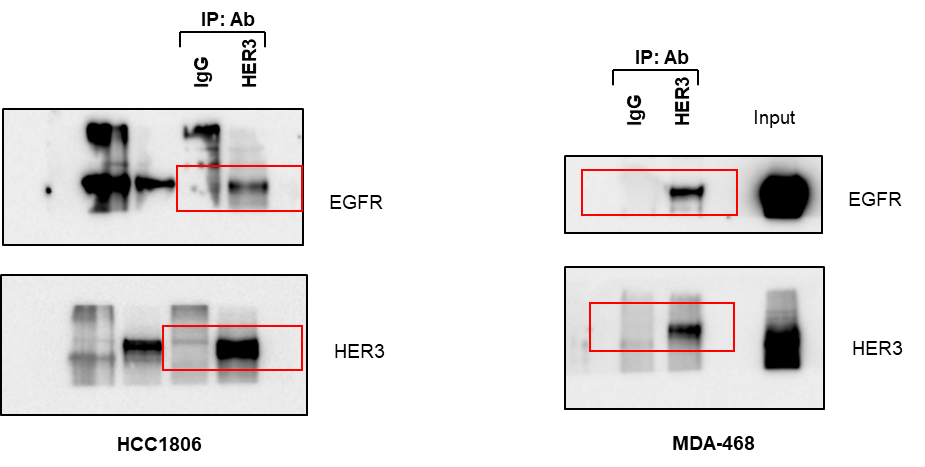


**Fig 4C(left)**


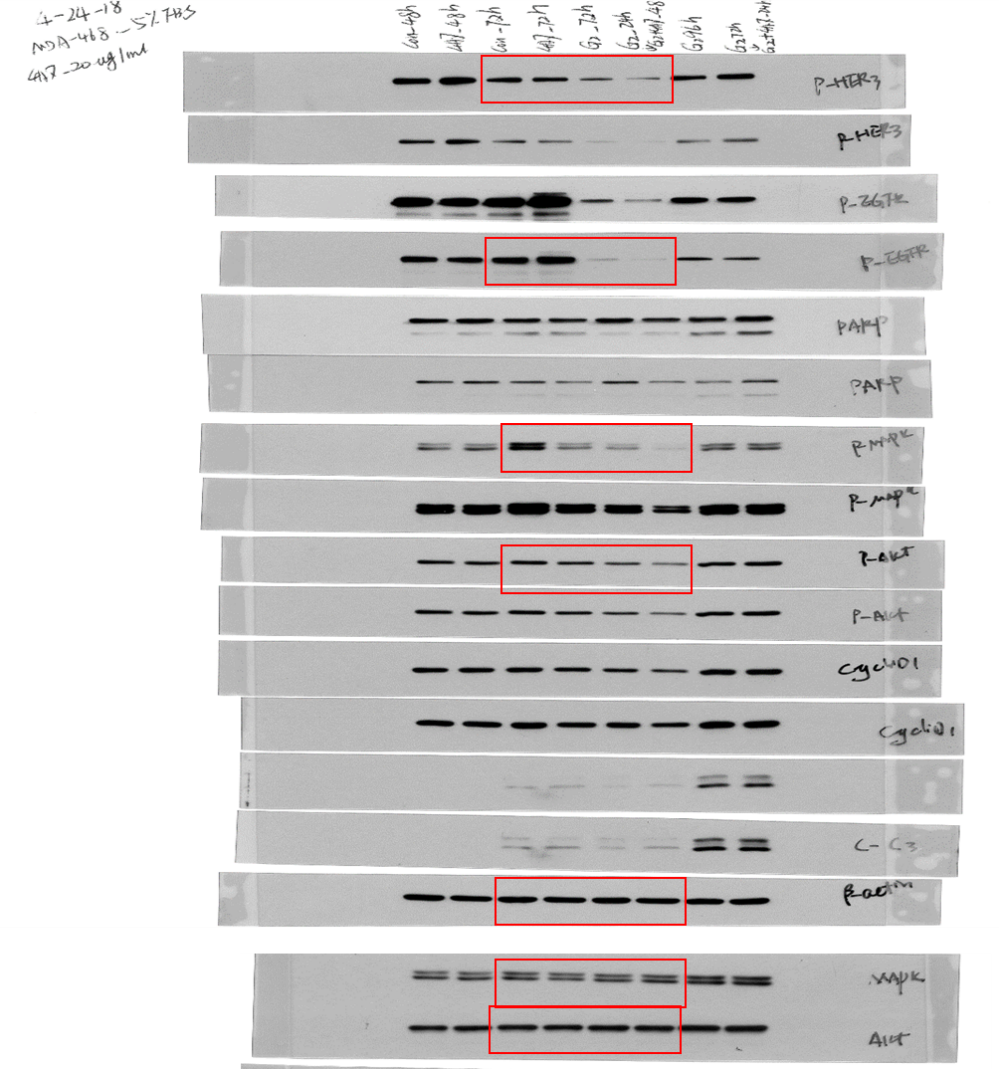


**Fig 4C(right)**


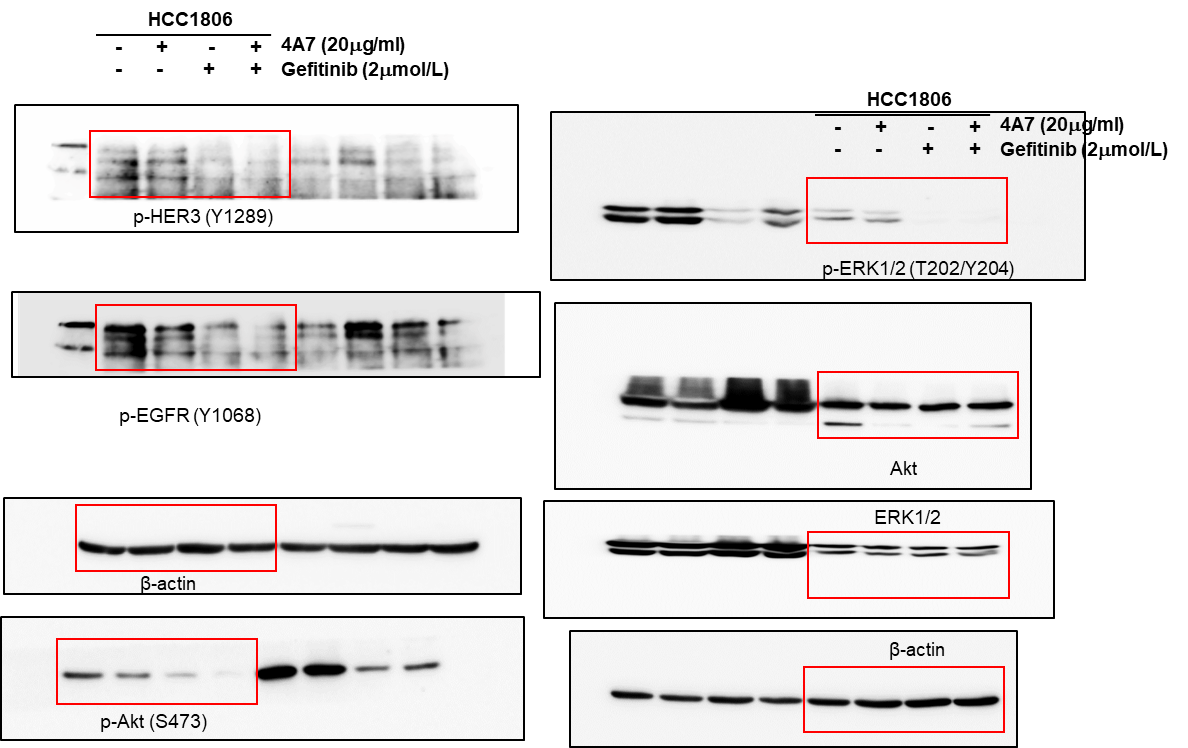


**Fig 5B(Left)**


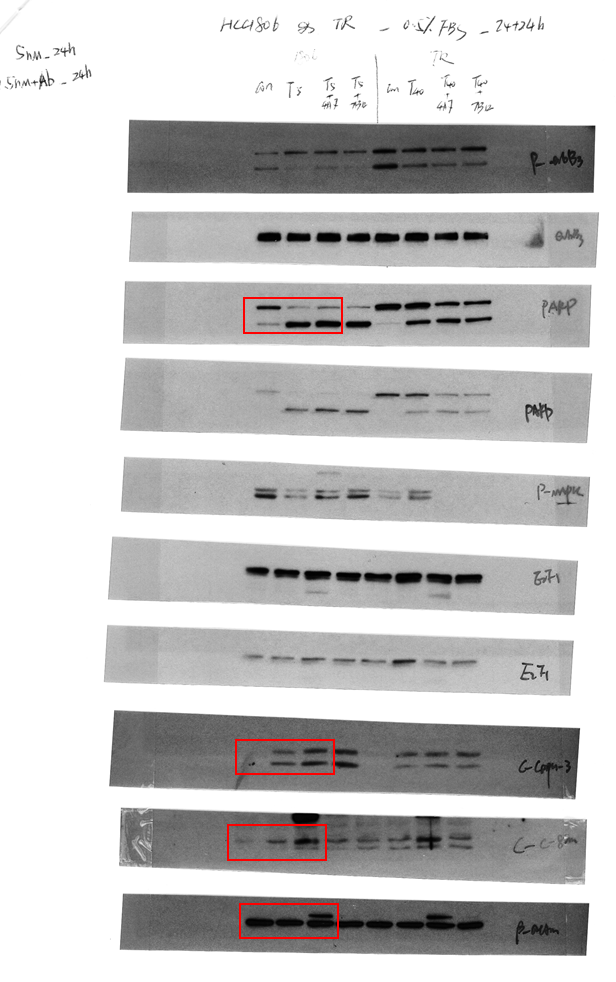

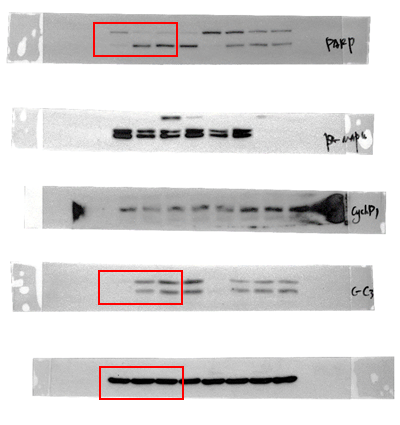


**Fig 5B (Right)**


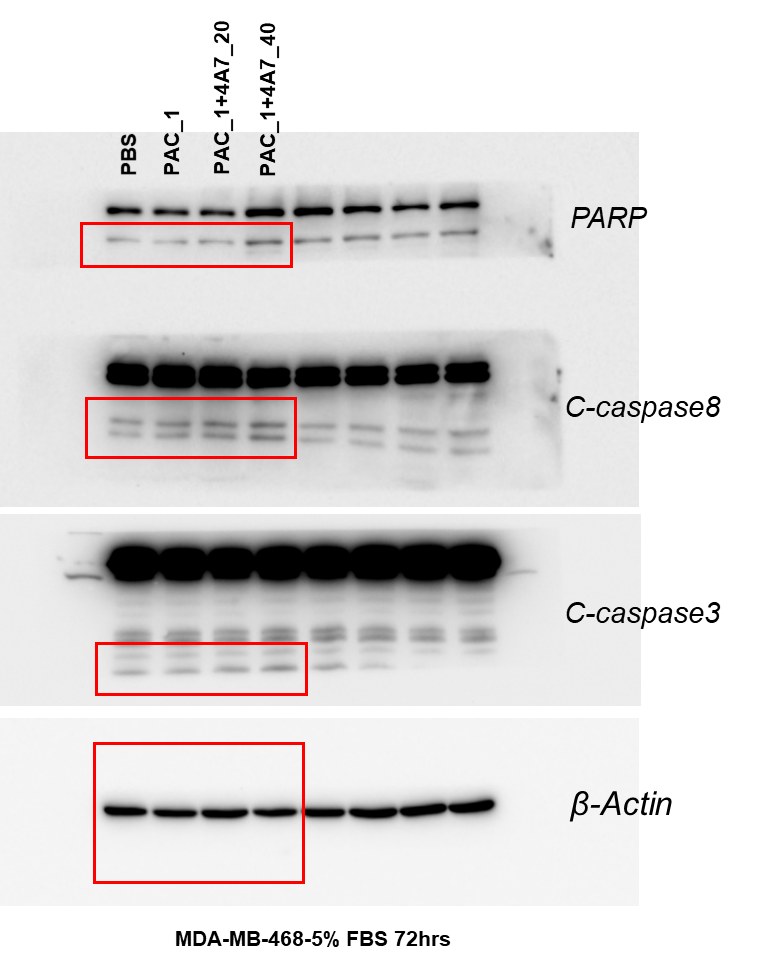


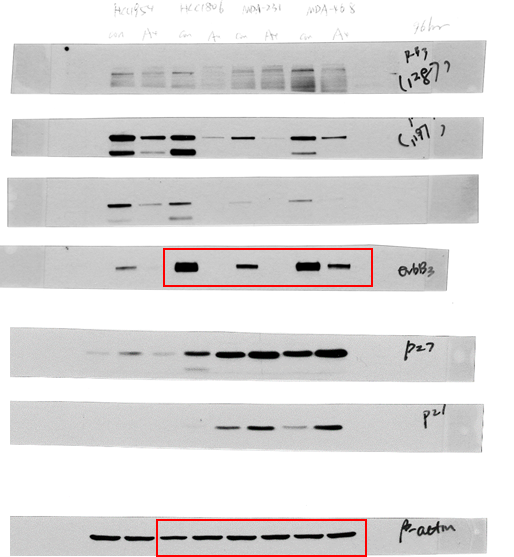


**Supplementary** Figure 1

Supplement: Supplementary file 2 — Supplementary Material 2 [file 12935_2023_3055_MOESM2_ESM.docx]
